# The REporting of studies Conducted using Observational Routinely-collected health Data (RECORD) Statement

**DOI:** 10.1371/journal.pmed.1001885

**Published:** 2015-10-06

**Authors:** Eric I. Benchimol, Liam Smeeth, Astrid Guttmann, Katie Harron, David Moher, Irene Petersen, Henrik T. Sørensen, Erik von Elm, Sinéad M. Langan, RECORD Working Committee

**Affiliations:** 1 Children’s Hospital of Eastern Ontario Research Institute, Department of Pediatrics and School of Epidemiology, Public Health and Preventive Medicine, University of Ottawa, Ottawa, Canada; 2 Institute for Clinical Evaluative Sciences, Toronto, Canada; 3 London School of Hygiene and Tropical Medicine, London, United Kingdom; 4 Hospital for Sick Children, Department of Paediatrics and Institute of Health Policy, Management and Evaluation, University of Toronto, Toronto, Canada; 5 Ottawa Hospital Research Institute, Ottawa, Canada, and School of Epidemiology, Public Health and Preventive Medicine, University of Ottawa, Ottawa, Canada; 6 Department of Primary Care and Population Health, University College London, London, United Kingdom; 7 Department of Clinical Epidemiology, Aarhus University, Aarhus, Denmark; 8 Cochrane Switzerland, Institute of Social and Preventive Medicine, University of Lausanne, Lausanne, Switzerland

## Abstract

Routinely collected health data, obtained for administrative and clinical purposes without specific a priori research goals, are increasingly used for research. The rapid evolution and availability of these data have revealed issues not addressed by existing reporting guidelines, such as Strengthening the Reporting of Observational Studies in Epidemiology (STROBE). The REporting of studies Conducted using Observational Routinely collected health Data (RECORD) statement was created to fill these gaps. RECORD was created as an extension to the STROBE statement to address reporting items specific to observational studies using routinely collected health data. RECORD consists of a checklist of 13 items related to the title, abstract, introduction, methods, results, and discussion section of articles, and other information required for inclusion in such research reports. This document contains the checklist and explanatory and elaboration information to enhance the use of the checklist. Examples of good reporting for each RECORD checklist item are also included herein. This document, as well as the accompanying website and message board (http://www.record-statement.org), will enhance the implementation and understanding of RECORD. Through implementation of RECORD, authors, journals editors, and peer reviewers can encourage transparency of research reporting.

## Introduction

The growing availability of data generated during health care delivery, and through monitoring of disease incidence and outcomes, has transformed the research landscape. Routinely collected health data are defined as data collected without specific a priori research questions developed prior to utilization for research [1]. These data sources could include provision of broad resources for research (e.g., disease registries), clinical management (e.g., primary care databases), health system planning (e.g., health administrative data), documentation of clinical care (e.g., electronic health record data repositories), or epidemiological surveillance (e.g., cancer registries and public health reporting data). These data, generated in various health care settings and geographic locations, present opportunities for innovative, efficient, and cost-effective research to inform decisions in clinical medicine, health services planning, and public health [2]. Internationally, governments and funding agencies have prioritized use of routinely collected health data as tools to improve patient care, transform health research, and improve health care efficiency [3].

While the explosion in data availability presents significant opportunities to answer pressing research questions, it also poses challenges for those undertaking and evaluating the research and implementing its findings. The broad spectrum of routinely collected health data sources and the rapid expansion of the field makes it challenging to identify the strengths and limitations and associated biases of individual data sources. Incomplete or inadequate reporting of research based on routinely collected data exacerbates these challenges. A systematic analysis of a sample of studies utilizing routine data sources has identified a variety of areas of incomplete or unclear reporting [4]. Reporting deficiencies include inadequate or missing information concerning coding of exposures and outcomes as well as details of linkage rates of different data sources. Two recent systematic reviews also document poor reporting of studies undertaken to validate data from routine data sources [5,6], which can obscure sources of bias, hamper efforts to undertake meta-analyses, and lead to erroneous conclusions.

**Fig 1:**
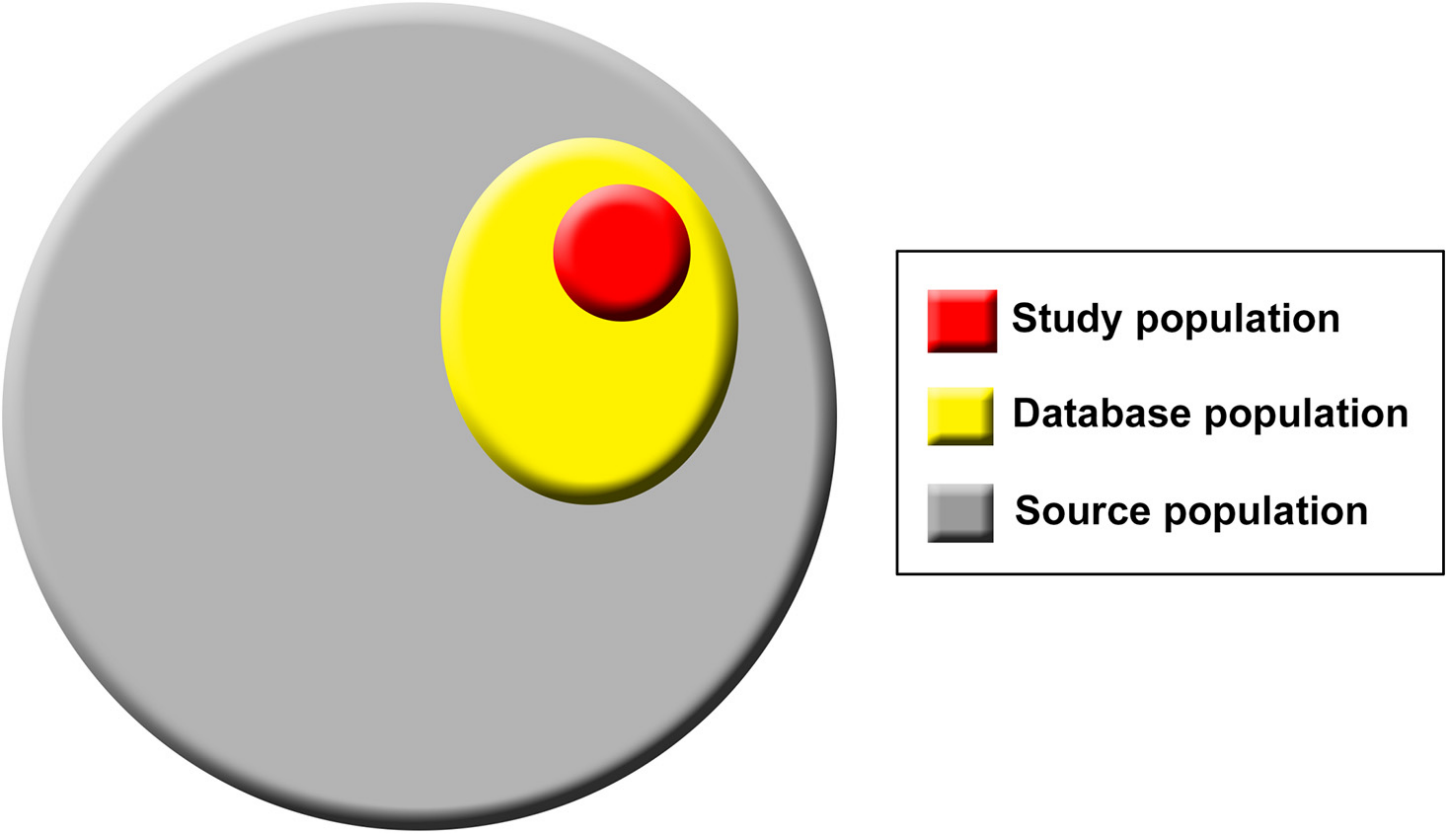
Population hierarchy in studies using routinely collected data sources.

Reporting guidelines have been developed to guide reporting for a range of study designs and contexts and are associated with improved quality of reporting [8,9]. The Strengthening the Reporting of Observational Studies in Epidemiology (STROBE) statement was developed to enhance the transparency of reporting of observational research [10,11] and has been widely adopted and endorsed by leading medical journals. It has been demonstrated to improve the quality of research reporting when implemented in the editorial process [12,13]. Most research conducted using routinely collected data is observational in design, and therefore, the STROBE guidelines are relevant and applicable. However, as the STROBE statement is designed to apply to all observational studies, specific issues related to reporting research using routinely collected data are not addressed. An international group of scientists with a specific interest in using routinely collected health data and representatives of the STROBE group met following the Primary Care Database Symposium in London in 2012 to discuss STROBE in the context of studies using routinely collected health data [14,15]. Important gaps in STROBE specific to research using these data sources were identified, and agreement was reached that an extension to STROBE was warranted. Thus, the REporting of studies Conducted using Observational Routinely collected Data (RECORD) initiative was established as an international collaborative process and an expansion of STROBE to explore and address specific reporting issues relevant to research using routinely collected health data. The RECORD initiative has involved more than 100 international stakeholders comprising researchers, journal editors, and consumers of data, including those utilizing research findings from routine data to inform decision making. The methodology used to develop the RECORD guidelines is detailed elsewhere [16] and based on the established methods to develop reporting guidelines [17]. In brief, stakeholders were surveyed twice to establish and prioritize themes for inclusion in the RECORD statement. A working committee then met in person to establish wording of the statements. Stakeholders reviewed the statements and provided feedback. The final checklist and this explanatory document were drafted by members of the steering committee, with review and approval by the working committee. Members of the STROBE steering committee were involved in the creation of RECORD.

Consistent with the STROBE approach, the RECORD guidelines are not designed to recommend the methods used to conduct research, but rather to improve its reporting to ensure that readers, peer reviewers, journal editors, and other consumers of research can assess its internal and external validity. By improving the quality of reporting of research using routinely collected health data, we seek to reduce unclear research reports and achieve the tenets of the scientific process: discovery, transparency, and replicability [18].

## Items in the RECORD Checklist

The complete RECORD checklist is provided in Table 1. Since RECORD is an extension to available STROBE items, statements are presented next to corresponding STROBE checklist items, organized by manuscript section. We advise authors to adequately address each item in the checklist but do not prescribe a precise order or location in the manuscript. Below we have provided explanatory text for each RECORD checklist item, organized by manuscript section. When no additional checklist items were required to expand STROBE to studies using routinely collected health data, any needed explanation is provided under the respective STROBE item.

**Table 1:** The RECORD statement: Checklist of items, extended from the STROBE statement, that should be reported in observational studies using routinely collected health data.

	Item Number	STROBE Items	RECORD Items
**Title and Abstract**			
	1	(a) Indicate the study’s design with a commonly used term in the title or the abstract. (b) Provide in the abstract an informative and balanced summary of what was done and what was found.	RECORD 1.1: The type of data used should be specified in the title or abstract. When possible, the name of the databases used should be included. RECORD 1.2: If applicable, the geographic region and time frame within which the study took place should be reported in the title or abstract. RECORD 1.3: If linkage between databases was conducted for the study, this should be clearly stated in the title or abstract.
**Introduction**			
Background rationale	2	Explain the scientific background and rationale for the investigation being reported.	
Objectives	3	State specific objectives, including any prespecified hypotheses.	
**Methods**			
Study Design	4	Present key elements of study design early in the paper.	
Setting	5	Describe the setting, locations, and relevant dates, including periods of recruitment, exposure, follow-up, and data collection.	
Participants	6	*(a) Cohort study*: Give the eligibility criteria and the sources and methods of selection of participants. Describe methods of follow-up. *Case-control study*: Give the eligibility criteria and the sources and methods of case ascertainment and control selection. Give the rationale for the choice of cases and controls. *Cross-sectional study*: Give the eligibility criteria and the sources and methods of selection of participants. *(b) Cohort study*: For matched studies, give matching criteria and number of exposed and unexposed. *Case-control study*: For matched studies, give matching criteria and the number of controls per case.	RECORD 6.1: The methods of study population selection (such as codes or algorithms used to identify subjects) should be listed in detail. If this is not possible, an explanation should be provided. RECORD 6.2: Any validation studies of the codes or algorithms used to select the population should be referenced. If validation was conducted for this study and not published elsewhere, detailed methods and results should be provided. RECORD 6.3: If the study involved linkage of databases, consider use of a flow diagram or other graphical display to demonstrate the data linkage process, including the number of individuals with linked data at each stage.
Variables	7	Clearly define all outcomes, exposures, predictors, potential confounders, and effect modifiers. Give diagnostic criteria, if applicable.	RECORD 7.1: A complete list of codes and algorithms used to classify exposures, outcomes, confounders, and effect modifiers should be provided. If these cannot be reported, an explanation should be provided.
Data sources/measurement	8	For each variable of interest, give sources of data and details of methods of assessment (measurement). Describe comparability of assessment methods if there is more than one group.	
Bias	9	Describe any efforts to address potential sources of bias.	
Study size	10	Explain how the study size was arrived at.	
Quantitative variables	11	Explain how quantitative variables were handled in the analyses. If applicable, describe which groupings were chosen and why.	
Statistical methods	12	(a) Describe all statistical methods, including those used to control for confounding. (b) Describe any methods used to examine subgroups and interactions. (c) Explain how missing data were addressed. (d) *Cohort study*: If applicable, explain how loss to follow-up was addressed. *Case-control study*: If applicable, explain how matching of cases and controls was addressed. *Cross-sectional study*: If applicable, describe analytical methods taking account of sampling strategy. (e) Describe any sensitivity analyses.	
Data access and cleaning methods		N/A	RECORD 12.1: Authors should describe the extent to which the investigators had access to the database population used to create the study population. RECORD 12.2: Authors should provide information on the data cleaning methods used in the study.
Linkage		N/A	RECORD 12.3: State whether the study included person-level, institutional-level, or other data linkage across two or more databases. The methods of linkage and methods of linkage quality evaluation should be provided.
**Results**			
Participants	13	(a) Report the numbers of individuals at each stage of the study (e.g., numbers potentially eligible, examined for eligibility, confirmed eligible, included in the study, completing follow-up, and analysed). (b) Give reasons for nonparticipation at each stage. (c) Consider use of a flow diagram.	RECORD 13.1: Describe in detail the selection of the persons included in the study (i.e., study population selection), including filtering based on data quality, data availability, and linkage. The selection of included persons can be described in the text and/or by means of the study flow diagram.
Descriptive data	14	(a) Give characteristics of study participants (e.g., demographic, clinical, and social) and information on exposures and potential confounders. (b) Indicate the number of participants with missing data for each variable of interest. (c) *Cohort study*: summarise follow-up time (e.g., average and total amount).	
Outcome data	15	*Cohort study*: Report numbers of outcome events or summary measures over time. *Case-control study*: Report numbers in each exposure category or summary measures of exposure. *Cross-sectional study*: Report numbers of outcome events or summary measures.	
Main results	16	(a) Give unadjusted estimates and, if applicable, confounder-adjusted estimates and their precision (e.g., 95% confidence interval). Make clear which confounders were adjusted for and why they were included. (b) Report category boundaries when continuous variables were categorized. (c) If relevant, consider translating estimates of relative risk into absolute risk for a meaningful time period.	
Other analyses	17	Report other analyses done—e.g., analyses of subgroups and interactions and sensitivity analyses	
**Discussion**			
Key results	18	Summarise key results with reference to study objectives.	
Limitations	19	Discuss limitations of the study, taking into account sources of potential bias or imprecision. Discuss both direction and magnitude of any potential bias.	RECORD 19.1: Discuss the implications of using data that were not created or collected to answer the specific research question(s). Include discussion of misclassification bias, unmeasured confounding, missing data, and changing eligibility over time, as they pertain to the study being reported.
Interpretation	20	Give a cautious overall interpretation of results considering objectives, limitations, multiplicity of analyses, results from similar studies, and other relevant evidence.	
Generalisability	21	Discuss the generalisability (external validity) of the study results.	
**Other Information**			
Funding	22	Give the source of funding and the role of the funders for the present study and, if applicable, for the original study on which the present article is based.	
Accessibility of protocol, raw data, and programming code		N/A	RECORD 22.1: Authors should provide information on how to access any supplemental information such as the study protocol, raw data, or programming code.

N/A, not applicable

### Title and Abstract

RECORD ITEM 1.1: The type of data used should be named in the title or abstract. Where possible, the names of the databases used should be included.

RECORD ITEM 1.2: If applicable, the geographic region and time frame within which the study took place should be reported in the title or abstract.

RECORD ITEM 1.3: If linkage between databases was conducted for the study, this should be clearly stated in the title or abstract.

#### Examples

Two examples of good reporting of this section are contained in the articles listed below:

1. “Perforations and Haemorrhages after Colonoscopy in 2010: A Study Based on Comprehensive French Health Insurance Data (SNIIRAM)” [19].
2. “The Dutch Hospital Standardised Mortality Ratio (HSMR) Method and Cardiac Surgery: Benchmarking in a National Cohort Using Hospital Administration Data versus a Clinical Database” [20].

#### Explanation

As there are no accepted Medical Subject Heading (MeSH) subject headings for identifying studies that use routinely collected health data, it is important to be able to identify a study as one conducted using such data. However, considering the wide variety of data types, simply stating that routine data were used is insufficient. Instead, the type of routine data should be specified in the title and/or abstract. Examples of data types include health administrative data, other administrative data (e.g., insurance, birth/death registries, and employment), disease registries, primary care databases, electronic health record data, and population registries. Naming the database(s) used is important but does not replace providing the type of data sources in the title or abstract.

The geographic region and time frame are items included in the STROBE checklist. We suggest that this information is also a necessary item in the title or abstract sections of manuscripts using the RECORD checklist. Clearly, the extent of reporting of region and time frame needs to adhere to word count limitations and take into account confidentiality issues. However, region should be reported at least at the largest geographical level used to define the study population (e.g., nation, state, province, and region).

In addition, linkage between databases (if it was conducted) should be reported in the title or abstract. Examples of acceptable wording include “using multiple linked health administrative databases” or “(database name) linked to (database name).” Using the words “linked” or “linkage” provides sufficient information in the title or abstract; further detail on linkage methodology should be provided in the Methods section of the manuscript.

### Introduction

No items specific to the RECORD guidelines are needed in addition to the STROBE items. The STROBE guidelines advise that “specific objectives, including any pre-specified hypotheses,” be stated in the Introduction section. Stating the specific research objectives is essential for replication and translation of any observational research. For studies using routinely collected data, authors should further clarify whether the analyses were exploratory with the purpose of finding new relationships in the data (examples are data mining or hypothesis-generating studies [21,22]) or confirmatory with the purpose of testing one or more hypotheses [23]. Authors also should indicate whether their hypotheses were generated before or after data analysis. They should clearly state whether there is a study protocol and how this can be accessed and if the study was registered in a publicly accessible study registry. Because the strengths and limitations of methods used to conduct research with routinely collected data may be contentious, a clear description of a study’s objectives is essential [23,24]. It is insufficient to simply label a study as descriptive without clarifying whether it aims to generate or examine a hypothesis.

### Methods (Setting)

No additional RECORD items are needed to expand the STROBE requirement to “describe the setting, locations, and relevant dates, including periods of recruitment, exposure, follow-up, and data collection.” Authors should note that beyond the type of database already alluded to in the title and/or abstract, information should be provided to allow the reader to understand the content and validity of the database and the original reasons why the data were collected. For instance, an electronic health record can be used by specialists or primary care physicians, for ambulatory or inpatient care, or by senior physicians or medical students. Users can be specifically trained for exhaustive and reproducible data entry, or no training may be provided [25]. Authors should also describe how the database population relates to the source population, including selection criteria, in order for readers to determine whether findings can be applied to the source population.

### Methods (Participants)

RECORD ITEM 6.1: The methods of study population selection (such as codes or algorithms used to identify subjects) should be listed in detail. If this is not possible, an explanation should be provided.

RECORD ITEM 6.2: Any validation studies of the codes or algorithms used to select the population should be referenced. If validation was conducted for this study and not published elsewhere, detailed methods and results should be provided.

RECORD ITEM 6.3: If the study involved linkage of databases, consider use of a flow diagram or other graphical display to demonstrate the data linkage process, including the number of individuals with linked data at each stage.

#### Examples

RECORD ITEM 6.1. An example of good reporting is provided in the following excerpt:

The OCCC [Ontario Crohn’s and Colitis Cohort] uses validated algorithms to identify patients with IBD based on age group. Each of these algorithms was validated in Ontario, in the specific age group to which it was applied, in multiple cohorts, medical practice types, and regions. For children younger than 18 years, the algorithm was defined by whether they underwent colonoscopy or sigmoidoscopy. If they had undergone endoscopy, children required 4 outpatient physician contacts or 2 hospitalizations for IBD within 3 years. If they had not undergone endoscopy, children required 7 outpatient physician contacts or 3 hospitalizations for IBD within 3 years.… This algorithm correctly identified children with IBD with a sensitivity of… [26].

This article referenced two previous validation studies of algorithms to identify patients with inflammatory bowel disease of different ages, including measures of diagnostic accuracy.

RECORD ITEM 6.2: 1. In their article, Ducharme and colleagues described in detail the validation of codes to identify children with intussusception and then used the validated codes to describe epidemiology. The codes involved in the validation study were listed in figure 2 of the article [27]. 2. In their article, Benchimol and colleagues did not conduct validation work; however, the validation work previously conducted was referenced. Details of diagnostic accuracy of identification algorithm codes were described [26].

RECORD ITEM 6.3: Some possible ways to illustrate the linkage process are demonstrated in the example figure 2, figure 3, and figure 4 on the RECORD website:

Figure 2. Venn diagram to illustrate linkage process (reproduced with permission from Herrett et al. [28] on our website: http://record-statement.org/images/figure2.jpg).

Figure 3. Mixed flow diagram and Venn diagram illustrating linkage process (reproduced with permission from van Herk-Sukel et al. [29] on our website: http://record-statement.org/images/figure3.jpg).

Figure 4. Linkage diagram combined with participant flow diagram (reproduced with permission from Fosbøl et al. [30] on our website: http://record-statement.org/images/figure4.jpg).

#### Explanation

RECORD ITEMS S 6.1 and 6.2: Reporting the validity of identification codes/algorithms used to derive the study population is essential to the transparency of reporting of observational research using routinely collected health data. In addition, reporting of codes/algorithms allows other investigators to engage in external or internal validation.

The methods used to identify study subjects should be explicitly and clearly stated, including whether identification is based on single codes, algorithms (combinations of records or codes), linkage between databases, or free-text fields.

The risk of misclassification bias in studies using routine health data, as in many other epidemiological studies, may threaten the validity of study findings [31]. Although the risk of misclassification is amplified in studies using databases containing large populations, such studies offer an opportunity to study rare or uncommon diseases [32]. Validation of identification methods has been increasingly emphasized as essential for studies using routinely collected health data, particularly for disease codes in studies using administrative data collected for billing purposes [33]. External validation studies typically entail comparing the codes or algorithms used to identify study populations to a reference standard. The most common reference standards are medical records, surveys of patients or practitioners, and clinical registries [5,34]. In addition, internal validation of databases may be undertaken to compare overlapping data sources within a single database [35]. Measures of accuracy are similar to those reported in diagnostic test studies, including sensitivity, specificity, positive and negative predictive values, or kappa coefficients [5,34].

Thus, for observational studies using routinely collected health data, we recommend that details of external or internal validation of identification codes/algorithms be presented in the Methods section of the manuscript. If one or more validation studies were previously undertaken, these should be referenced. If such validation studies were not conducted, this should be explicitly stated. In addition, a brief discussion of the accuracy of the identification methods (using common diagnostic accuracy terms) and their functioning in the subpopulations under study should be included. If validation work was conducted as part of the observational study in question, we suggest that authors use the published reporting guidelines for validation studies [5]. It is important to state whether the validation occurred in a source or database population different from that selected for the present study, as codes may function differently in different populations or databases [36]. In addition, if there are known problems with the reference standard to which the data were compared, for example, incompleteness or inaccuracy, these problems should be reported and additionally be discussed as a limitation. Authors should discuss the implications of using the selected codes/algorithms to identify study populations and outcomes, the risk of misclassification, and the potential impacts on study findings. It is particularly important to discuss the implications of relying on a validation study conducted in a population different from the one being examined.

RECORD ITEM 6.3: A flow diagram or other graphical display can convey useful information about the linkage process and can simplify a potentially lengthy description. Such illustrations can provide key data such as information on the proportion and characteristics of the linked and unlinked individuals. Readers should be able to establish the proportion of the database populations that were successfully linked and the representativeness of the resulting study population. Linkage flow diagrams can either be stand-alone diagrams (e.g., Venn or flow diagrams) or can be combined with the participant flow diagram as recommended by STROBE. As graphical displays can be provided in many formats, we do not recommend a specific one.

### Methods (Variables)

RECORD ITEM 7.1: A complete list of codes and algorithms used to classify exposures, outcomes, confounders, and effect modifiers should be provided. If these codes or algorithms cannot be reported, an explanation should be provided.

#### Examples

1. Hardelid and colleagues provided all codes in their S1 Table in Data Supplement 2 [37].
2. Murray and colleagues provided all codes for at-risk groups in their Appendix S1 [38].

#### Explanation

Just as with codes/algorithms used to identify the study population, codes/algorithms to classify exposures, outcomes, confounders, or effect modifiers subject the research to potential misclassification bias. In order to allow for replication, evaluation, and comparisons to other studies, we recommend that a list of all the diagnostic, procedural, medication, or other codes used to conduct the study be provided in the manuscript, an online appendix, and/or an external website. For routine data consisting of survey results, the survey questions should be provided with the precise wording given to study subjects. Considering the risk of misclassification bias in all research, including research conducted using routine health data [31], authors should provide sufficient detail to make their research reproducible and to make the risk of bias apparent. Validation studies may be described in the article manuscript or provided as references to other published or online material. As noted above, authors should state whether the validation study was conducted in a source or database population different from that examined in the present study.

We recognize that in some situations, researchers may be prevented from providing code lists and algorithms used in a publication, as this information is considered proprietary or protected by copyright, intellectual property, or other laws. For example, some comorbidity adjustment indices have been created by for-profit companies and sold to researchers for use in academic research settings [39,40]. In these situations, authors may have relied on data providers or trusted third parties to collect, clean, and/or link the data. Authors should provide a detailed explanation regarding their inability to provide code lists or other details on how individuals or conditions are identified and should endeavour to include contact information for the group holding proprietary rights to these lists. In addition, authors should address how their inability to provide this information may impact consumers of the research in terms of research replication and evaluation. Optimally, the third parties should provide detailed information on how the data were collected, cleaned, or linked. Improved communication between data providers and data users could be mutually beneficial.

Some have argued that code lists represent the researchers’ intellectual property. Publication of these lists could allow other researchers to use them for their own research, thereby depriving the authors of their intellectual property and credit for creating the code list. We felt that this view is inconsistent with the scientific standard of transparency to allow for replication of research. Therefore, apart from those protected by law or contract, we recommend that the full code lists be published.

Considering word count and space restrictions in many journals and the potential length of code lists/algorithms, we recognize that publication in a paper-format journal article may not be possible. Instead, detailed information could be reported in the text, published tables, online supplements on the journal website as appendices, hosted online permanently by the authors or other individuals, or deposited in a third party data repository (e.g., Dryad or Figshare). The text and reference sections of the manuscript should provide detailed information on how to access code lists. Code repositories such as ClinicalCodes.org hold great promise for the documentation and transparency of codes used in research based on health data [41]. If the code lists are published in online supplements on the journal website or on an external website provided by the authors, the link should be published in the main journal article. Publication on a journal website or on PubMed Central (http://www.ncbi.nlm.nih.gov/pmc/) increases the probability that the supplement will be available as long as the journal is operational. If publication on an external private or institutional website is the only option, we recommend that these lists continue to be available for at least 10 years following publication of the journal article. If the URL address is changed, automatic redirection from the old web address is required. These measures will allow for future readers of the article to have access to the complete code lists.

In addition to code lists provided in the article (or an online appendix), the authors should include a reflection on whether the choice of codes/algorithms used in the study might lead to bias. Such bias could include misclassification bias, ascertainment bias, and bias due to missing data. If sensitivity analyses were conducted based on different sets of codes/algorithms, these should also be described and evaluated. Discussion of potential bias could also be linked to other parts of the RECORD and STROBE checklists, such as study subject selection, and validation of codes (or lack thereof).

### Methods (Statistical Methods)

#### Data access and cleaning methods

RECORD ITEM 12.1: Authors should describe the extent to which the investigators had access to the database used to create the study population. RECORD ITEM 12.2: Authors should provide information on the data cleaning methods used in the study.

#### Linkage

RECORD ITEM 12.3: State whether the study included person-level, institutional-level, or other data linkage across two or more databases. Linkage techniques and methods used to evaluate linkage quality should be provided.

#### Examples

RECORD ITEM 12.1: The following articles describe access to a subset of the UK General Practice Research Database (GPRD).

1. “The GPRD restricts its data sets to 100,000 individuals for projects funded through the Medical Research Council licence agreement. This restriction mandated a case-control rather than cohort design to ensure we identified sufficient cases of cancer for each particular symptom…” [42].
2. “A random sample from the General Practice Research Database…was obtained under a Medical Research Council licence for academic institutions [43].

RECORD ITEM 12.2: The following is an example of a data cleaning methods description [44]:

Completeness of common identifiers for linking varied between datasets and by time (identifiers were more complete in recent years). For LabBase2, completeness of identifiers varied by unit (figure 2). For PICANet [Paediatric Intensive Care Audit Network], date of birth and hospital number were 100% complete, and the majority of other identifiers were >98% complete, with the exception of NHS [National Health Service] number (85% complete). For both datasets, cleaning and data preparation were undertaken: NHS or hospital numbers such as “Unknown” or “9999999999” were set to null; generic names (e.g., “Baby,” “Twin 1,” “Infant Of”) were set to null; multiple variables were created for multiple surname and first names; postcodes beginning “ZZ” (indicating no UK postcode) were set to null.

RECORD ITEM 12.3: The following excerpts from articles are good examples of good reporting of the level of data linkage, the linkage techniques and methods used, and the methods used to evaluate linkage quality:

1. “We linked live birth and fetal death certificates into chronological chains of events that, excluding induced abortions and ectopic pregnancies, constituted the reproductive experience of individual women” [45].
2. Two articles contain excellent descriptions of linkage undertaken specifically for the study being reported [44,45]. In the article by Harron and colleagues [44], a detailed explanation on linkage is provided with graphical demonstration of the match process. In addition, the methods to calculate probability of linkage are described: “Match probabilities P(M|agreement pattern) were calculated to estimate the probability of a match given agreement on a joint set of identifiers. This avoided the assumption of independence between identifiers. Probabilities were derived as the number of links divided by the total number of pairs for each agreement pattern (based on probable links identified in the training datasets). For example, if 378 comparison pairs agreed on date of birth and Soundex but disagreed on sex, and 312 of these were probable links, the match probability for the agreement pattern [1,1,0] was 312/378 = 0.825” [44]. The article by Adams and colleagues also provided a detailed explanation of the linkage process: “The deterministic linkage consisted of phase I, which entailed six processing steps during which chains were formed and individual (previously unlinked) records were added to chains. Next followed phase n, which entailed multiple passes through the file to combine chains belonging to the same mother” [45].
3. By contrast, if a study refers to prior linked data, referring to a prior paper may be adequate as follows: “Records from both databases were linked to the municipal registries based on date of birth, gender and zip code, and were subsequently linked to each other. The linkage was performed by Statistics Netherlands and is described in previous publications” [20].
4. The following is an example of good reporting of characteristics of linked and unlinked individuals: “For the purposes of this paper unmatched ISC [Inpatient Statistics Collection] records will be referred to as ISC residuals, unmatched MDC [Midwives Data Collection] records as MDC residuals and linked pairs as matched records….Selected variables that were available on both data sets were compared across three groups—ISC residuals, MDC residuals and matched records” [46].

#### Explanation

RECORD ITEMS 12.1 and 12.2: Errors can occur if data analysts unfamiliar with the nuances of cohort creation or study aims create the study cohorts. Consequently, the extent authors had access to the database should be reported. The description of data cleaning methods at different stages of the study should include those used to screen for erroneous and missing data, including range checks, checks for duplicate records, and handling of repeated measures [47,48]. Other methods to be reported could include assessment of frequency distributions and data cross tabulations and graphical exploration or use of statistical methods for outlier detection [49]. Further detail could be provided on error diagnosis, including definitions of plausibility, and error handling in the analysis. A clear and transparent description of data cleaning methods is important, as choice of methods could affect study findings, repeatability of the study, and reproducibility of study findings [50].

RECORD ITEM 12.3: For linkage studies, we suggest reporting on the estimated rate of successful linkage, use of deterministic versus probabilistic linkage, quality and type of variables used for linkage, and results of any linkage validation. If linkage of records across databases was conducted specifically for the study, methods of linkage and linkage quality evaluation should be reported, including information on who performed the linkage. As available, details should be provided on blocking variables, completeness of linkage variables, linkage rules, thresholds, and manual review [44]. If linkage was conducted prior to the study (i.e., for previous studies or for general use) or if data linkage was undertaken by an external provider, such as a data linkage centre, then a reference is needed describing the data resource and linkage methods.

Data describing linkage methods and evaluating their success are critical to permit the reader to assess the impact of any linkage error and related bias [51]. Specifically, the reader should know whether the type of linkage used was deterministic and/or probabilistic, in order to determine whether linkage could be affected by false matches or missed matches. Deterministic linkage is useful when a unique identifier is available across the different data sources. When such an identifier is unavailable, a description of the record linkage rules applied (or statistical linkage keys) is critical. In contrast, probabilistic linkage uses multiple identifiers, sometimes with different weights, and matches are considered present above a specific threshold. Mixed methods also may be used. For instance, deterministic linkage may be used for some records, and probabilistic linkage may be applied when unique identifiers are unavailable for other records. Linkage bias occurs when associations are present between the probability of linkage error (e.g., false and missing matches) and the variables of interest. For example, linkage rates may vary by patient characteristics, e.g., age, gender, and health status. Even small errors in the linkage process can introduce bias and lead to results that can overestimate or underestimate the associations under study [52]. Authors should report linkage error using standard approaches including comparisons with gold standards or reference datasets, sensitivity analyses, and comparing characteristics of linked and unlinked data [53]. Reporting linkage error allows the reader to determine the quality of the linkage and the possibility of bias related to linkage error.

### Results (Participants)

RECORD ITEM 13.1: Describe in detail the selection of the persons included in the study (i.e., study population selection), including filtering based on data quality, data availability and linkage. The selection of included persons can be described in the text and/or by means of the study flow diagram.

#### Example

An example of good reporting is given in the following excerpt:

We identified 161,401 Medicare beneficiaries given a diagnosis of one or more cases of cancer of the lung and bronchus in the SEER [Surveillance, Epidemiology, and End Results] registries between 1998 and 2007. Among these patients, we identified a total of 163,379 separate diagnoses of incident lung cancer. (Some patients had two cases of primary lung cancer separated by more than a year during the study period). Fig 1 shows the derivation of the final cohort of 46,544 patients with 46,935 cases of NSCLC [non-small cell lung cancer] [54]. (See figure 5 for the example flow diagram, available at http://record-statement.org/images/figure5.jpg.)

#### Explanation

The authors should provide a clear description of the derivation of the study population(s) from the original database of routinely collected health data, as differences between the study population and the database population need to be documented to enable application of the results (See also RECORD item 6.1). Researchers using routine data sources frequently limit their study population based on factors such as the quality of available data. For example, they may restrict the study period to a time when the data quality is known to be acceptable, resulting in the exclusion of potential participants. Studies may exclude medical practices with inconsistent electronic health record entry or wait for those practices to become consistent [38,55]. The study population also may be restricted based on data availability. For example, in studies utilising United States Medicare data, beneficiaries currently registered in a health maintenance organisation are frequently excluded because of lack of records of clinical events [54,56]. When using data sources in which eligibility fluctuates over time (e.g., insurance databases) researchers need to specify clearly how eligibility was defined and how changes in eligibility were managed in their study. If a study utilises linked routine data, the study population is frequently reduced through restriction to individuals for whom linked data are available [57]. Highly restricted cohorts may also be used for methodological reasons to eliminate some sources of confounding.

Thus, steps taken to derive the final study population(s), inclusion and exclusion criteria, and inclusion and exclusion of study participants at different stages in cohort creation and analysis should be clearly defined in the manuscript, either in the text or using a suitable flow diagram. Study populations may be derived using different codes and/or algorithms (see RECORD item 6.1), and different use of codes over time may impact the study population [58,59]. Some studies may also have used several case definitions that are more or less sensitive/specific, which may have an impact on subsequent analyses. Delineation of these steps is important in assessing the external validity of study findings and, in certain circumstances, assessing possible selection bias. Sensitivity analyses may be reported to evaluate the potential impact of missingness of data and representativeness of the study population. Providing information on selection of the study population(s) from the initial database also permits the study to be replicated. Subsidiary analyses may have been performed on different study populations and may potentially be reported in online appendices.

### Discussion (Limitations)

RECORD ITEM 19.1: Discuss the implications of using data that were not created or collected to answer the specific research question(s). Include discussion of misclassification bias, unmeasured confounding, missing data, and changing eligibility over time, as they pertain to the study being reported.

#### Examples

The following papers describe limitations associated with the use of administrative data:

1. “Third, this study was a retrospective, claims-based analysis. Only PET [positron emission tomography] scans paid for by Medicare could be detected in the analysis. To minimize the proportion of missed claims, all analyses were limited to Medicare beneficiaries with both Medicare Part A and Part B coverage and no enrollment in managed care or Medicare Part C for the 12 months before and after diagnosis. Fourth, patients in the SEER registry are more likely to be nonwhite, to live in areas with less poverty, and to live in urban areas, which may limit the generalizability of the findings. Fifth, during the study period, disease stage was based on SEER data obtained over 4 months or until first surgery. In 2004, data collection for SEER changed to the collaborative staging system. It is unclear how our results would differ with this newer approach” [54].
2. “Despite several strengths of the SEER-Medicare data, including a comparatively large sample size, generalizability to the US population, and detailed information on prescriptions, our study was limited by the lack of laboratory data on cholesterol, triglyceride, and glucose levels that would have informed the extent of metabolic disturbances in the population…thus having laboratory-based data could have reduced residual confounding by severity of metabolic disease. We also lacked more granular data on cancer progression, which could have confounded the association between statin use and death, given that statin treatment may be withheld or discontinued in patients with short expected survival time” [60].

#### Explanation

Routine health data are not typically collected with a specific a priori research question in mind, and the reasons motivating the data collection may vary. Numerous potential areas of bias, including all the usual sources of bias associated with observational research but also some more specific to observational research using routine data, endanger the conclusions of researchers. The following should be discussed by authors as potential sources of bias: (1) codes or algorithms to identify study populations, outcomes, confounders, or effect modifiers (misclassification bias); (2) missing variables (unmeasured confounding); (3) missing data; and (4) changes in eligibility over time.

The rationale underlying routine data collection may affect the quality and applicability of the data to research questions being examined. For example, registries used for retrospective analyses may implement better quality control than organizations collecting other types of routine data, although this may vary. Similarly, some administrative data are subject to careful quality control, while other data are not. Administrative data are particularly subject to errors in upcoding or opportunistic coding. For example, when hospital reimbursement is based on complexity of the case mix, hospitals may maximize reimbursement by liberally applying more complex disease codes to patient records [61]. In addition, changes in coding strategies may impact validity or consistency of data. For example, the introduction of provider billing incentive codes may change the likelihood of a code being used over time [62,63]. Other codes may be avoided because of patient stigmatization or provider penalties [64]. In addition, changes in versions of code classification systems (e.g., from International Classification of Diseases (ICD)-9 to ICD-10) may alter the validity of ascertainment using coded data [65,66]. Variation in clinical practice across hospitals and populations may result in laboratory investigations being undertaken in specific locations and/or practices, which may impact a diagnostic algorithm. If any of these potential sources of misclassification bias are present, they should be discussed as study limitations.

Unmeasured confounding is defined as confounding associated with variables not included in the data under study, leading to residual confounding bias [67]. While it is a potential source of bias in all observational research, it is particularly prominent in studies using routinely collected data. The analysis may require variables that were not considered when the databases were being planned or when the data were collected. A variety of methods have been proposed to address this potential source of bias [68–71], including propensity scores. However, propensity score analyses, like standard regression analyses and matching, can only guarantee a balance of study participants on variables that are available in the data. A particular type of unmeasured confounding is confounding by indication; this is often an issue when examining effectiveness and safety of (drug) treatments using routinely collected data. Hence, the prognosis of those receiving the (drug) treatment may be better or worse than those who are not, but information on prognosis and/or severity of the underlying illness may not be available in the data [72]. Such issues should be discussed by the authors, and the methods used to take this into account (when possible) should be reported.

Missing data are problematic for all observational research and have been addressed in Box 6 of the STROBE explanatory article [10]. Missing data are a particular issue for routinely collected data, as researchers cannot control data collection [73]. Missing data can result in selection bias if there are missing values in variables used to define the study cohort or missing identifiers that prevent records from being linked, particularly if the missing data occur nonrandomly. Missing variables create similar challenges. Authors should delineate the missing variables suspected of causing unmeasured confounding, the reason these variables were missing, how this may have affected study results, and the methods used to adjust for missing variables. For example, smoking status has a strong effect on Crohn’s disease severity and has been associated with outcomes of this disease. However, smoking status is rarely included in health administrative data. In a study assessing the association between socioeconomic status and Crohn’s disease outcomes, smoking status was discussed as a potential unmeasured confounding factor [74]. Frequently, missing data/missing variables are discovered only after initiation of research using routine health data, making it necessary for investigators to deviate from their original research protocol. Details of deviation from the protocol, irrespective of the reason for deviation, should always be reported. Reasons for the deviation and the implications on the research and conclusions should be discussed.

Another important potential limitation is changes in coding practices or eligibility criteria resulting from a change in the composition of the database population, study population, or both over time. The definition of the database population may change under a number of circumstances, e.g., if enrolling practices cease collaboration with the database, change computer software, or change criteria for enrolment in the database change, such as a registry. The study population in administrative data sources (e.g., insurance databases) may change if persons’ eligibility is not constant over time, because of changes in employment, residency status, or medical care provider. A change in the way records are coded (e.g., upcoding or changes in coding systems, as described above) may alter the study population [63,75,76]. When discussing limitations, authors should explain how changing eligibility was handled in the analysis so the reader can assess the potential for bias. As detailed by STROBE, the discussion should include the direction and magnitude of any potential bias and efforts taken to address such bias.

### Other Information

RECORD ITEM 22.1: Authors should provide information on how to access any supplemental information such as the study protocol, raw data, or programming code.

#### Examples

1. The article by Taljaard and colleagues represents the full research protocol for a study using the Canadian Community Health Survey [77].
2. In their article, Guttmann and colleagues invite requests for the study protocol: “Data sharing: The technical appendix, dataset creation plan/protocol, and statistical code are available from the corresponding author at [email address]” [78].

#### Explanation

We strongly support the dissemination of detailed information on study methods and results. When possible, we encourage the prior or simultaneous publication of the study protocol, raw data results, and, if applicable, programming code. This information is useful to peer reviewers and readers in assessing the validity of study findings. A number of opportunities are available to researchers for open publication of such data. These include online journal supplementary material, personal websites, institutional websites, science-based social media sites (e.g., ResearchGate.net and Academia.edu), data repositories (e.g., Dryad or Figshare), or government open data websites [79]. We recognize that some research organizations, corporations, institutions, or laws may prohibit or restrict the free availability of such information. While a discussion of ownership and use of this intellectual property is outside the scope of the RECORD guidelines, posting of such data should always be performed within the legal and ethical guidelines of the researchers’ institutional environment, with the guidance of journal editors. This information would also be useful to other researchers who may wish to access these data to replicate, reproduce, or expand upon the research described in the manuscript. Whatever the format or extent of available supplemental information, we recommend that reference to the location of this information be clearly stated in the manuscript.

## Discussion

The RECORD guidelines are specific to observational research conducted using routinely collected health data and serve to supplement, not replace, the STROBE guidelines. RECORD was created as a guide for authors, journal editors, peer reviewers, and other stakeholders to encourage transparency and completeness of reporting of research conducted using routinely collected health data. The checklist is intended for use by any researcher using such data, and we encourage wide dissemination to all interested parties. We anticipate that endorsement and implementation of RECORD by journals will improve the transparency of reporting of research using routinely collected health data.

### Limitations

Both STROBE and RECORD are intended for application only to observational research studies. However, routinely collected health data are sometimes used for research conducted with other study designs, such as cluster-randomized trials for health system evaluation. In addition, linkage of data from randomized trials to administrative data can be used for long-term follow-up of outcomes, and associated studies would not be considered observational. As the field evolves, we expect to expand RECORD to other research designs using similarly rigorous methods.

While RECORD represents our best attempt to reflect the interest and priorities of stakeholders, we recognize that the methods used to conduct research using routinely collected health data are changing rapidly, and the availability of types of data for such research is expanding. For example, mobile health applications (mHealth apps) are becoming widely available for smartphones and wearable technologies. While limited research is presently conducted using these data sources, we anticipate rapid growth in the use of these data in the near future, and new methodologies will be created to manage this resource. In addition, the working committee decided to focus on health data, and not on all data sources used to conduct health-related research (e.g., environmental data, financial data, etc.). Therefore, the RECORD checklist may not reflect themes that will become important in the future, and revision may be necessary at some point.

Extensive efforts were made to include a broad representation of stakeholders in the creation of these guidelines. We recruited stakeholders through open calls and targeted invitations using a variety of channels [16]. However, stakeholder representation was predominantly from regions conducting research using routinely collected health data, with only a few representatives from developing nations and non-English speaking countries. Nevertheless, we believe that the stakeholder group was representative of the current community of researchers and users of the generated knowledge. While a great deal of input was obtained through surveys and feedback from the stakeholder group, feasibility dictated that the statements were crafted by a smaller working committee consisting of 19 members who met in person, as previously suggested in the literature [17]. In the future, technology and social media may allow for more active participation by larger groups in working committee meetings.

### Future Directions and Community Engagement

As the availability of routinely collected health data expands, we expect more involvement of researchers from regions in which such data are not currently accessible. Through the record-statement.org website and message board, we expect ongoing commentary and discussion on the RECORD document from interested parties, which may result in official revisions in the future. Through this online community, RECORD will become a living document that can adapt to changes in the field.

Publication of a reporting guideline and endorsement by journals are not sufficient to improve research reporting [80]. The manner in which the guidelines are implemented by researchers, journals, and peer reviewers are of key importance to RECORD having a measurable impact [81]. Therefore, the online message board will include a discussion forum on implementation. We also encourage assessment of the impact of RECORD on reporting in the field to ensure that the guidelines provide measurable benefit.

## Conclusions

The RECORD statement expands the STROBE criteria to observational studies conducted using routinely collected health data. With the input of the research and publishing community, we have created reporting guidelines in the form of a checklist and this accompanying explanatory document. Reporting guidelines have been demonstrated to improve reporting of research, thereby allowing for consumers of the research to be aware of the strengths, limitations, and accuracy of conclusions [12,82–84]. While we anticipate that RECORD will change with the evolution of research methods in the field, these guidelines will help facilitate adequate reporting of research over the coming years. With implementation by authors, journal editors, and peer reviewers, we anticipate that RECORD will result in transparency, reproducibility, and completeness of reporting of research conducted using routinely collected health data.

**Box 1. Definitions of Population Terms (Source, Database, and Study Populations).**

There are three levels of population hierarchy that are relevant in studies using routinely collected data and will be referred to throughout the statement. These populations include the source population, which represents that from which the database population is derived and hence about which the researchers want to make inferences; the database population, which is derived from the source population and comprises people with data included in the data source; and the study population, identified from within the database population by the researchers using codes and algorithms (Fig 1) [7]. For example, in the case of the Clinical Practice Research Datalink (CPRD), the source population comprises all people attending general practitioners in the United Kingdom. The database population comprises those individuals included in CPRD, while the study population comprises those selected from within CPRD using codes and algorithms to be described in the specific study.

## Supporting Information

S1 Table List of stakeholders who participated in the surveys. (XLSX)

## Abbreviations

CPRD: Clinical Practice Research Datalink
GPRD: General Practice Research Database
HSMR: hospital standardised mortality ratio
ICD: International Classification of Diseases
ISC: New South Wales Inpatient Statistics Collection
MDC: New South Wales Midwives Data Collection
MeSH: Medical Subject Heading
mHealth apps: mobile health applications
NHS: National Health Service
NSCLC: non-small cell lung cancer
PET: positron emission tomography
PICANet: Paediatric Intensive Care Audit Network
RECORD: REporting of studies Conducted using Observational Routinely collected health Data
SEER: Surveillance, Epidemiology, and End Results
SNIIRAM: Système national d'informations inter régimes de l'Assurance maladie
STROBE: Strengthening the Reporting of Observational Studies in Epidemiology
